# Perceptions of poverty in Spain: differences in the attitudinal profiles between women and men

**DOI:** 10.3389/fpsyg.2023.1229685

**Published:** 2023-11-15

**Authors:** M. Carmen Terol Cantero, Maite Martin-Aragón Gelabert, Carolina Vázquez Rodríguez, Ana Lledó Boyer, Jose Enrique García Soler

**Affiliations:** ^1^Department of Behavioral Sciences and Health, University Miguel Hernández, Elche, Spain; ^2^Department of Methodology, National University of Distance Education, Cartagena, Spain

**Keywords:** attitudes, stereotypes, poverty, profiles, women

## Abstract

Poverty is a multidimensional phenomenon that encompasses privation of education, health or housing. Women show more positive perceptions of poor people, making external attributions for the causes of poverty or the circumstances that explain it. The aim of this study is to analyse perceptions of poverty, identifying the differences in attitudinal profiles between women and men, and the influence of their political and religious beliefs. The sample consists of 278 participants (154 women and 124 men), who completed a sociodemographic questionnaire and the Scale of Attitudes and Stereotypes toward Poverty. The results showed two attitude profiles for women and men, with differences in the first profile, where women or men did not have religious beliefs, had left-wing or centre-left political ideas and favourable attitudes about poverty.

## Introduction

Poverty is a multi-dimensional phenomenon that encompasses privation of education, health, or housing (Bayón, 2013), and is a relevant factor in social vulnerability. According to The World Bank (2022), by the end of 2022, as many as 685 million people (9.3% of the world population) could still be living in extreme poverty, suffering severe difficulties to satisfy their most basic needs, such as health, education, and access to water and sanitation. In Spain, 27.8% of the population lives in poverty and social exclusion (European Anti-Poverty Network, 2022). In macro-economic terms, reports warn of an increase in unemployment, leading to an increase in poverty and vulnerability (European Anti-Poverty Network, 2022). In this socio-economic context, the negative perceptions of the poor and their functioning warrant our understanding, since they underlie responses and behaviours regarding social inequalities, justice, equity, and social policy development (e.g., Mayorga Coy, 2018; Bastias et al., 2019; Contreras-Montero and Hidalgo-Mesa, 2021). This interest has led to the development of many studies on the perception and causal explanations or attitudes are the most frequently constructs researched (Dakduk et al., 2010; Lepianka et al., 2010; Pereira Da Costa and Dias, 2013; Sainz et al., 2022).

Currently, approach of attitudinal models has converged in a definition that contemplates a global evaluative disposition toward attitude, which is determined by components or experiences of cognitive, affective and behavioural information in relation to the attitudinal object and is inferred from observable, cognitive, affective and behavioural or behavioural intention responses (Bohner and Wanke, 2002; López-Sáez et al., 2019). Within this framework, social perception processes contemplate the emotional or affective component as a fundamental element of our attitudes toward people and groups, which can predict our behaviour as well as our relationship with others and the world. In turn, the cognitive component of attitude or stereotype, which is generated through our social interaction, is configured as a culturally defined mental representation, and is therefore subject to social, ideological and cultural determinants (Vázquez and Martínez, 2008).

## Women’s perception of poverty

There is a link between the social construction of gender and the mental representation of either stereotype, which can also be expressed through the attitudes that distinguish men and women in relation to a social phenomenon or group, as in the case of poverty. Some studies show, for example, how a culturally established gender system can privately and publicly influence women’s perceptions and the activation of a stereotype (Quinn et al., 2003; Vázquez and Martínez, 2011). These different perceptions of poverty depend on three factors that construct the internal representation of gender: social practices and roles, social representations of gender, and gender inequalities (Vázquez and Martínez, 2012). In this sense, different studies highlight that it is women who show more favourable beliefs, referring to external factors as causes of poverty and that there are unpredictable or uncontrollable circumstances that lead the poor to be in this situation (Norcia et al., 2010; Bastias et al., 2019). According to this social and cultural construction of gender, other studies confirm that women’s perspective of this problem goes beyond individual circumstances; they also display more favourable attitudes toward the poor and are more in favour of welfare policies (Norcia et al., 2010; Bastias et al., 2019).

## Determinants of poverty perception

Related to the perception of poverty, social class or socio-economic status has been considered a relevant determinants of perceptions of poverty, indicating that people from a low social class or low socio-economic level tend to perceive poverty as linked to external factors. Conversely, those from an upper social class or socio-economic level relate it to the internal or dependent factors of an individual (Norcia et al., 2010; Mickelson and Hazlett, 2014; Bastias et al., 2019). In addition, values like political and religious beliefs are also shown to influence the perceptions and attitudes toward poverty. For those with a more conservative stance or having right-wing or centre-right ideas, more individual or personal responsibility explanations are referred to. On the other hand, those with a more progressive stance or with left-wing or centre-left ideas emphasize the structural and social aspects and have more favourable attitudes toward the welfare system (Bobbio et al., 2010; Lepianka et al., 2010; Vázquez et al., 2017; Boeh, 2018; Bergmann and Todd, 2019; Contreras-Montero and Hidalgo-Mesa, 2021; Toft and Calhoun, 2021; Vázquez and Panadero, 2022). With respect to religion, some studies do not agree that there is a relation between traditional or catholic beliefs and situational or external explanations for the causes of poverty (Vázquez et al., 2017), while other studies document this result (Lepianka et al., 2010). In contrast, other studies link these religious beliefs to factors of individual responsibility for poverty (Boeh, 2018; Bergmann and Todd, 2019).

## The present study

In sum, evidence on the perception of poverty supports the relationship of the aforementioned variables, such as being a woman, or social class, as well as the role of other individual political and religious beliefs. But, this study emphasizes the importance of differentiating the reactions of women and men within their cultural context with their religious values and ideologies, which can explain the more favourable attitudes of women toward social welfare, vulnerable groups and engaging in activities volunteering (Wemlinger and Berlan, 2016; Bellindo et al., 2021). Therefore, the aim of this study is to analyse perceptions of poverty, identifying the differences in attitudinal profiles between men and women, and the influence of their political and religious beliefs. In line with literature, the hypotheses are: (1) that women will show more favourable attitudes and attitudinal profiles toward poverty; and (2) that individuals with more favourable attitude profiles will differ in their political ideas (left-wing or centre-left) and perception of belonging to a low or middle-low social class.

## Method

### Participants

The sample consisted of 278 participants in higher education: 154 women and 124 men (44.6%) (mean age: 21.59 ± 5.3 years; Range = 18–53). In this group, 42.8% (*n* = 119) self-identified as belonging to the upper or middle-upper class and 57.2% (*n* = 159) to the lower or middle-lower class. Regarding religious beliefs, 56.5% indicated none and 43% indicated having them. In political beliefs, most participants (65%) defined themselves as left wing or centre left. The sample of women and men did not differ in age (*z* = − 1.52; *p* = 0.13), perceived social class (*X*^2^ = 2.53; *p* = 0.12; *V* = 0.09; *p* = 11) or political ideas (*X*^2^ = 1.10; *p* = 0.29; *V* = 0.07; *p* = 29), but did differ in religious beliefs, which were more frequent in women (*X*^2^ = 4.19; *p* = 0.04; *V* = 0.12; *p* = 0.04).

### Instruments

#### Sociodemographic characteristics

Information about age, sex, education level and self-perceived social class (upper or upper-middle-class and lower-middle or lower class) were collected.

#### Religious and political beliefs

They were measured with two *ad-hoc* items: “According to your *religious* or *political* beliefs, which do you identify with?” Participants chose from four options. For religious beliefs: None, Catholicism, Islamism, Other. For political beliefs: left wing, centre left, centre right or right wing. The construction of these items is based on studies carried out by Lepianka et al. (2010) and Bergmann and Todd (2019).

#### Attitudes toward poverty

The affective component of attitude toward poverty was evaluated using the *Attitudes Towards Poverty Scale* by Cozzarelli et al. (2001). This scale includes 12 items and a total score indicates how favourable their attitude is toward poverty. Responses are given on a Likert scale of 1 to 5, where one means “totally disagree” and 5 “totally agree.” Internal consistency: *α* = 83; *ω* = 0.84.

To evaluate the cognitive component of attitude toward poverty, the *Stereotypes about the Poor Scale* was used (Cozzarelli et al., 2001). This instrument comprises 38 items: 15 refer to positive stereotypes, and 23 refer to negative stereotypes. Responses are given on a Likert scale of 1–5, where 1 means “not at all characteristic of poor people” and 5 “extremely characteristic of poor people.” Internal consistency index for the Positive Stereotypes subscale: *α* = 0.83 y *ω* = 0.85; and for the Negative Stereotypes subscale: *α* = 0.92 y *ω* = 0.93.

### Procedure and data analysis

This is a cross-sectional study with a convenience non-probabilistic sample. Participants took part voluntarily and completed the questionnaires in a self-administered paper version or recorded version via Google Form. All of them were informed about the aim of this project, confidentiality and anonymity by a researcher. They signed the informed consent.

IBM SPSS v.28 software was used for statistical analysis. Means and frequencies were used for the descriptive analyses. The Kolmogorov–Smirnov test was carried out for normality distribution. The Mann–Whitney U test was used for difference analysis, and effect size was calculated using the r-Rosenthal index (Rosenthal, 1991): small effect size (0.10), medium effect size (0.30) and large effect size (0.50) (Rosenthal, 1991). Contingency tables were used for difference analyses in the case of categorical variables. The hierarchical cluster analyses using the Ward method was used to determine the different psychosocial profiles related to attitudes toward poverty. In addition, the data scores were normalised so that the scale for each variable was the same. Once the profiles were established, the mean values of the variables in each cluster and the effect size of these differences were compared.

## Results

### Differences between women and men

For the *affective component* of attitude toward poverty, the sample shows favourable attitudes toward poor people (*M* ± sd:3.96 ± 0.50; *M* ± sd:4.10 ± 0.49; men and women, respectively). Women generally have a more positive attitude toward poverty than men (*z* = −2.35; *p* = 0.019; *r*_bis_ = 14). Women differ significantly from men, since their *feelings towards poor people are generally positive* (z = −2.72; *p* = 0.007; *r*_bis_ = 0.16) and they are more *concerned about poor people* (*z* = −4.13; *p* ≤ 0.001; *r*_bis_ = 0.25), with effect sizes being between small and medium. In addition, women give stronger disagree responses for items like the following: *I do not like poor people very much* (z = −2.23; *p* = 0.26; *r*_bis_ = 0.13).

In the *cognitive component* of attitude or stereotypes, the lower mean scores refer to characteristics of negative stereotyping (men *M* ± sd: 2.42 ± 0.56; women *M* ± sd: 2.32 ± 0.62). In both, negative and positive stereotyping, there are no significant differences between men and women. However, although the effect size is small, women considered it more characteristic for poor people to be *intelligent* (*z* = −1.89; *p* = 0.05; *r*_bis_ = 0.11) or *weak* (*z* = −2.18; *p* = 0.030 *r*_bis_ = 0.13), and less characteristic for them to be *proud* (*z* = −2.72; *p* = 0.007; *r*_bis_ = 0.16), *uneducated* (*z* = −2.63; *p* = 0.009; *r*_bis_ = 0.16), *abusive* (*z* = −2.26; *p* = 0.024; *r*_bis_ = 0.14), *unkind* (*z* = −2.02; *p* = 0.043; *r*_bis_ = 0.12), *promiscuous* (*z* = −2.85; *p* = 0.004; *r*_bis_ = 0.17) or *inconsiderate* (*z* = −1.92; *p* = 0.05; *r*_bis_ = 0.12; Table 1).

**Table 1:** Differences between women and men in attitudes toward poverty.

Attitude-affective	Women (*M* ± sd)	Men (*M* ± sd)	*z*	*p*	*r*_bis_
I do not like poor people very much	1.46 ± 0.86	1.65 ± 0.81	−2.23	0.026	0.13
My feelings toward poor people are generally positive	3.73 ± 0.82	3.49 ± 0.72	−2.72	0.007	0.16
I am concerned about poor people	3.80 ± 0.77	3.40 ± 0.81	−4.13	<0.001	0.25
I feel that poor people are worthy of respect	4.69 ± 0.67	4.57 ± 0.75			
Affective-total	4.10 ± 0.49	3.96 ± 0.50	−2.35	0.019	0.14
Attitude-positive stereotype					
Proud	2.25 ± 1.03	2.49 ± 1.03	−2.72	0.007	0.16
Intelligent	3.16 ± 0.65	2.99 ± 0.72	−1.89	0.05	0.11
Positive stereotype-total	3.09 ± 0.49	3.09 ± 0.48	n.s.	0.473	–
Attitude – negative stereotype					
Uneducated	2.16 ± 1.01	2.50 ± 1.02	−2.63	0.009	0.16
Weak	2.34 ± 1.09	2.03 ± 0.85	−2.18	0.030	0.13
Abusive	1.89 ± 0.90	2.12 ± 0.86	−2.26	0.024	0.14
Unkind	1.80 ± 0.83	2.02 ± 0.90	−2.02	0.043	0.12
Promiscuous	1.95 ± 0.92	2.30 ± 0.93	−2.85	0.004	0.17
Inconsiderate	2.05 ± 0.95	2.28 ± 0.95	−1.92	0.05	0.12
Negatives stereotype – total	2.32 ± 0.62	2.42 ± 0.56	n.s.	0.099	–

*M*, Mean; SD, Standard Deviation; *r*_bis_, rank-biserial correlation; *p*, significance level.

### Profiles of women and men

Two profiles are obtained for women. The first defines 75% of the sample, showing characteristics that differ from the second profile (25%). These are low or middle-low social class (*p* = 0.020), with no religious beliefs, and following left-wing or centre-left political ideas (*p* < 0.001). This profile of women shows more favourable perceptions about poor people and differs significantly from the second profile in the affective component (*p* = 0.024) and the cognitive component of attitude (positive and negative stereotype) (*p* < 0.01), with a small-medium effect size (Table 2).

**Table 2:** Profiles of women.

	Cluster 1 (*n* = 87)	Cluster 2 (*n* = 28)	*χ* ^2^	(*V*) *p*	
	%	%			
Social class	34/53^1^	18/10^1^	5.43	(0.22) 0.020	
Religious beliefs	36/51^2^	22/6^2^	11.72	(0.32) <0.001	
Political beliefs	8/79^3^	28/0^3^	81.22	(0.84) <0.001	

	*M* ± sd	*M* ± sd	*z*	*p*	*r*_bis_
Affective-attitude	4.15 ± 0.51	3.87 ± 0.50	−2.26	0.024	0.21
Positive stereotype-attitude	3.19 ± 0.44	2.89 ± 0.38	−2.69	0.007	0.25
Negative stereotype-attitude	2.18 ± 0.59	2.56 ± 0.56	−2.89	0.004	0.27

^1^Upper or Upper-Middle-class and Lower-Middle or Lower class; ^2^Si/No; ^3^Left, Centre-left, Centre-right and the Right; *χ*^2^, chi square; *V* de Cramer for nominal variables; *p*, bilateral significance.

The analysis of men’s attitudes toward poverty also shows two profiles. The first represents 72% of the sample, which differs significantly from the second profile (28%). The majority see themselves as not religious, with left-wing or centre-left ideas, with a more favourable attitude and a more positive stereotype of poor people (*p* < 0.001), showing a medium-large effect size (Table 3).

**Table 3:** Profiles of men.

	Cluster 1 (*n* = 73)	Cluster 2 (*n* = 28)			
	%	%	*χ* ^2^	(*V*) *p*	
Social class	28/45^1^	13/15^1^	0.46	(0.074) 0.46	
Religious beliefs	13/60^2^	27/1^2^	52.3	(0.720) <0.001	
Political beliefs	18/55^3^	21/7^3^	21.7	(0.461) <0.001	

	*M* ± sd	*M* ± sd	*z*	*p*	*r*_bis_
Affective-attitude	4.14 ± 0.42	3.45 ± 0.41	−5.817	<0.001	0.55
Positive Stereotype –Attitude	3.17 ± 0.51	2.86 ± 0.39	−3.450	<0.001	0.33
Negative Stereotype-Attitude	2.26 ± 0.54	2.81 ± 0.39	−4.414	<0.001	0.42

^1^Upper or Upper-Middle-class and Lower-Middle or Lower class; ^2^Si/No; ^3^Left, Centre-left, Centre-right and the Right; *χ*^2^, chi square; *V* de Cramer for nominal variables; *p*, bilateral significance.

**Figure 1:**
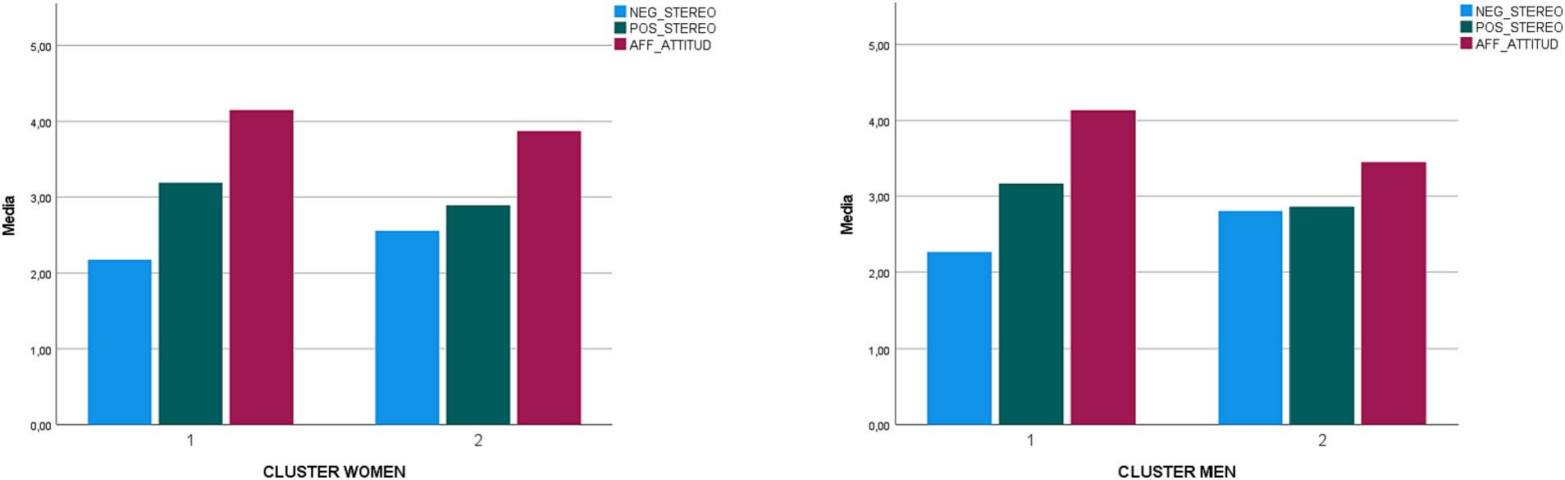
Graphical representation of the two clusters in Women and Men.

Figure 1 illustrates the profiles of women and men’s attitudes toward poverty. The analysis of the differences between women and men in the first profile is not significant for either the affective component of attitude (*z* = −3.38; *p* = 0.74), or the cognitive component: positive stereotype (*z* = −0.52; *p* = 61) and negative stereotype (*z* = −0.99; *p* = 0.32). In the comparison between women and men in profile 2, there are no differences for the cognitive component of attitude: positive stereotype (*z* = −0.82; *p* = 0.41) and negative stereotype (*z* = −1.39; *p* = 17). However, women do show a more favourable affective attitude than men (*z* = −3.73; *p* < 0.001).

## Discussion

In a first general analysis of the differences between women and men, women show more positive affective attitudes toward poor people, which is in line with the reviewed studies on the perception of poverty (Bullock, 1999; Norcia et al., 2010; Bastias et al., 2019). However, the results do not present significant differences in the cognitive component or stereotype (positive or negative). Women’s stereotype of the poor is especially characterized by a lower negative loading, and according to the proposals of attitudinal theories (Osgood and Tannenbaum, 1955; Festinger, 1957; Heider, 1958), this would “coherently” lead women’s attitude to be more positively affective in contrast to men.

Within profile analysis, both women and men present two profiles. One matches to the majority (≥0.70%) and is characterized by more favourable affective attitudes, higher positive stereotype and less negative stereotype, and no religious beliefs as well as left-wing or centre-left political ideas. The second profile (≥0.25%) comprises those who present less favourable attitudes, a less positive and more negative stereotype, with religious beliefs and more right-wing or centre-right ideologies. It is important to note that a middle-large effect size is obtained for the difference between the first and second profiles of men, whereas it is small-middle for women. In addition, there is a difference with respect to social class in women, being mainly low or middle-low as in the first profile, which represents more favourable attitudes. In the comparison between women and men, in profiles 1 and 2, no differences were found in the cognitive component (positive and negative stereotype). With respect to the affective component, there were no differences in the profiles 1, but significant differences were found in the profiles 2, where women showed more positive affective attitudes than men (*z* = −3.73; *p* < 0.001). Nevertheless, this group of women does show less favourable affective attitudes (*z* = −2.26; *p* = 0.024) compared with the women in the first profile (low or middle-low class, no religious beliefs, left-wing or centre-left political ideas).

Thus, for the first hypothesis, women confirmed positive attitudinal differences in the affective component. According to the results of the profiles, these differences demonstrate an effect mediated by the processes of socialisation developed around gender roles and stereotypes but in interaction with certain religious or political values. Knowing all the complexity of these processes would further define the cognitive and attitudinal development of women in private and public contexts, and how their attitudes toward poverty and the poor also determine their commitments to equality or social justice (Quinn et al., 2003; Vázquez and Martínez, 2011, 2012; Özpinar and Akdede, 2022; Vázquez and Panadero, 2022). Nevertheless, in view of the results, this mediation of religious and political values and their interaction with gender could also reveal the differences between women’s profiles and between men’s profiles.

For the second hypothesis, the influence of both women and men’s political ideas is confirmed. This supports the results of previous research about poverty and political ideas (Bullock, 1999; Ljubotina and Ljubotina, 2007; Bobbio et al., 2010; Lepianka et al., 2010; Vázquez et al., 2017; Boeh, 2018; Bergmann and Todd, 2019; Contreras-Montero and Hidalgo-Mesa, 2021; Toft and Calhoun, 2021; Vázquez and Panadero, 2022). However, according to the profile analysis for social class, we found that its influence on attitudes is confirmed for women only. This result differs from most of the literature reviewed regarding the importance attributed to social class in perceptions of poverty (Bullock, 1999; Cozzarelli et al., 2001; Lepianka et al., 2010; Norcia et al., 2010; Mickelson and Hazlett, 2014; Pereira Da Costa and Dias, 2015; Bastias et al., 2019; Yúdica et al., 2021; Mdluli and Dunga, 2022).

## Conclusion

The results of this study provide a reflection on the influence of religious and ideological values through socialisation processes, and an understanding of differential cognitive and affective development according to contexts and learning. Since stereotypes and perceptions about poverty play an important role in supporting the implementation of welfare policies and social programs for poor people, this study confirms that women show positive attitudes toward poverty depending on different religious or political values (Vázquez et al., 2017; Morias et al., 2019; Vázquez and Panadero, 2022). Given this, it is important to note that the process of socialisation across ideologies and religious or spiritual values may be even more powerful than campaigns designed to change attitudes toward poverty and toward the social welfare system in the community.

### Limitations of the study

First, this study used convenience sampling, represented primarily by youth, in higher education making it difficult to be able to compare with other older age groups,or different educational levels. In this study, social class was assessed according to its own perception, however, it would be of interest to use objective indicators, such as income, occupation, among others. With respect to beliefs, extreme and moderate political ideas have been combined but analysing each groups from different positions would enrichen the outcomes regarding the influence of these political ideas on the development of the attitudes evaluated. Lastly, religious beliefs are overrepresented by Catholicism, but other religious or spiritual beliefs could broaden the explanation of how these values determine particular attitudes.

## Data availability statement

The raw data supporting the conclusions of this article will be made available by the authors, without undue reservation.

## Ethics statement

The studies involving humans were approved by Research ethics and integrity committee. Vicerrectorate of Research Miguel Hernández University of Elche (O.I.R: Research Office Registered: 191204114702; 2019.546.E.OIR; Reference: DCC.MMG.01.19). The studies were conducted in accordance with the local legislation and institutional requirements. The participants provided their written informed consent to participate in this study.

## Author contributions

MM-A and MT: conceptualization. MT and JG: methodology and formal analysis. MT: data curation and supervision. AB and CV: writing—original draft preparation. CV and MT: writing—review and editing. CV: visualisation. MM-A: project administration. All authors have read and agreed to the published version of the manuscript.
